# Supplementary material for: Economic Burden of Not Complying with Canadian Food Recommendations in 2018
Source: Nutrients. 2019 Oct 20;11(10):2529. doi: 10.3390/nu11102529 (PMC6835951; doi:10.3390/nu11102529)
Supplement: Supplementary file 1 [file nutrients-11-02529-s001.zip › Supplementary materials/S2 Table_Aug21.docx]

**S2 Table:** Percentages of the 2015 Canadian population ≥2 years by age and sex, and by level of food intake

**Nuts and Seeds Servings (svgs)**

Females

|  | <0.5 svgs/d | 0.5-<1 svgs/d | ≥1 svg/d |
| --- | --- | --- | --- |
| ≤14 years | 97.8% | 1.3% | 1.0% |
| 15-34 years | 94.2% | 3.1% | 2.7% |
| 35-54 years | 91.7% | 4.3% | 4.1% |
| 55-64 years | 89.5% | 5.0% | 5.6% |
| 65-74 years | 92.1% | 5.2% | 2.8% |
| ≥75 years | 94.5% | 3.3% | 2.2% |

Males

|  | <0.5 svgs/d | 0.5-<1 svgs/d | ≥1 svg/d |
| --- | --- | --- | --- |
| ≤14 years | 97.4% | 2.1% | 0.5% |
| 15-34 years | 94.5% | 0.8% | 4.7% |
| 35-54 years | 90.4% | 3.3% | 6.3% |
| 55-64 years | 91.6% | 4.5% | 4.0% |
| 65-74 years | 94.1% | 2.9% | 3.0% |
| ≥75 years | 93.9% | 3.6% | 2.6% |

**Whole Grain Servings (svgs)**

Females

|  | <0.5 svgs/d | 0.5-<1 svgs/d | 1-<1.5 svgs/d | 1.5-<2 svgs/d | ≥2 svgs/d |
| --- | --- | --- | --- | --- | --- |
| ≤14 years | 33.4% | 35.7% | 19.9% | 7.9% | 3.2% |

|  | <0.5 svgs/d | 0.5-<1 svgs/d | 1-<1.5 svgs/d | 1.5-<2 svgs/d | 2-<2.5 svgs/d | 2.5-<3 svgs/d | ≥3 svgs/d |
| --- | --- | --- | --- | --- | --- | --- | --- |
| 15-34 years | 45.8% | 22.2% | 14.2% | 8.3% | 4.6% | 2.5% | 2.5% |
| 35-54 years | 46.7% | 22.4% | 13.6% | 8.3% | 4.5% | 2.4% | 2.2% |
| 55-64 years | 48.1% | 23.0% | 13.6% | 7.7% | 4.1% | 2.0% | 1.6% |
| 65-74 years | 35.5% | 23.4% | 16.8% | 11.1% | 6.5% | 3.6% | 3.3% |
| ≥75 years | 31.2% | 25.3% | 18.6% | 11.8% | 6.6% | 3.7% | 2.9% |

Males

|  | <0.5 svgs/d | 0.5-<1 svgs/d | 1-<1.5 svgs/d | 1.5-<2 svgs/d | ≥2 svgs/d |
| --- | --- | --- | --- | --- | --- |
| ≤14 years | 31.8% | 32.9% | 20.4% | 9.6% | 5.3% |

|  | <0.5 svgs/d | 0.5-<1 svgs/d | 1-<1.5 svgs/d | 1.5-<2 svgs/d | 2-<2.5 svgs/d | 2.5-<3 svgs/d | 3-<3.5 svgs/d | 3.5-<4 svgs/d | ≥4 svgs/d |
| --- | --- | --- | --- | --- | --- | --- | --- | --- | --- |
| 15-34 years | 40.8% | 21.2% | 13.8% | 9.2% | 6.2% | 3.9% | 2.2% | 1.3% | 1.4% |
| 35-54 years | 41.3% | 21.0% | 13.8% | 9.3% | 6.2% | 3.6% | 2.2% | 1.3% | 1.3% |

|  | <0.5 svgs/d | 0.5-<1 svgs/d | 1-<1.5 svgs/d | 1.5-<2 svgs/d | 2-<2.5 svgs/d | 2.5-<3 svgs/d | 3-<3.5 svgs/d | ≥3.5 svgs/d |
| --- | --- | --- | --- | --- | --- | --- | --- | --- |
| 55-64 years | 42.6% | 21.7% | 13.8% | 8.9% | 5.9% | 3.2% | 1.8% | 2.1% |
| 65-74 years | 30.0% | 20.9% | 15.9% | 11.9% | 8.3% | 5.3% | 3.5% | 4.2% |
| ≥75 years | 32.5 | 20.9 | 16.8 | 12.2 | 7.6 | 4.6 | 2.5 | 2.8 |

**Fruit Servings (svgs)**

Females

|  | <0.5 svgs/d | 0.5-<1 svgs/d | 1-<1.5 svgs/d | 1.5-<2 svgs/d | ≥2 svgs/d |
| --- | --- | --- | --- | --- | --- |
| ≤14 years | 13.0% | 22.1% | 22.5% | 17.3% | 25.1% |

|  | <0.5 svgs/d | 0.5-<1 svgs/d | 1-<1.5 svgs/d | 1.5-<2 svgs/d | 2-<2.5 svgs/d | 2.5-<3 svgs/d | ≥3 svgs/d |
| --- | --- | --- | --- | --- | --- | --- | --- |
| 15-34 years | 28.4% | 20.4% | 17.2% | 13.6% | 9.1% | 5.3% | 6.1% |
| 35-54 years | 23.6% | 20.1% | 18.4% | 14.4% | 10.3% | 6.2% | 7.1% |
| 55-64 years | 20.7% | 19.0% | 18.4% | 15.4% | 10.9% | 6.9% | 8.8% |
| 65-74 years | 21.0% | 20.2% | 18.8% | 15.6% | 10.8% | 6.5% | 7.3% |
| ≥75 years | 19.8% | 21.3% | 20.9% | 16.0% | 10.3% | 5.9% | 5.9% |

Males

|  | <0.5 svgs/d | 0.5-<1 svgs/d | 1-<1.5 svgs/d | 1.5-<2 svgs/d | ≥2 svgs/d |
| --- | --- | --- | --- | --- | --- |
| ≤14 years | 13.6% | 22.6% | 22.3% | 17.1% | 24.4% |

|  | <0.5 svgs/d | 0.5-<1 svgs/d | 1-<1.5 svgs/d | 1.5-<2 svgs/d | 2-<2.5 svgs/d | 2.5-<3 svgs/d | ≥3 svgs/d |
| --- | --- | --- | --- | --- | --- | --- | --- |
| 15-34 years | 34.6% | 20.3% | 15.6% | 11.5% | 7.9% | 4.7% | 5.4% |
| 35-54 years | 29.0% | 19.9% | 16.5% | 13.1% | 9.0% | 5.7% | 6.7% |
| 55-64 years | 25.9% | 19.3% | 16.9% | 13.5% | 9.8% | 6.6% | 8.0% |
| 65-74 years | 25.9% | 19.8% | 17.6% | 13.6% | 9.7% | 6.1% | 7.3% |
| ≥75 years | 24.5% | 21.3% | 19.1% | 14.6% | 9.4% | 5.4% | 5.7% |

**Vegetable Servings (svgs)**

Females

|  | <0.5 svgs/d | 0.5-<1 svgs/d | 1-<1.5 svgs/d | 1.5-<2 svgs/d | 2-<2.5 svgs/d | 2.5-<3 svgs/d | ≥3 svgs/d |
| --- | --- | --- | --- | --- | --- | --- | --- |
| ≤14 years | 14.1% | 30.1% | 24.0% | 14.6% | 8.3% | 4.3% | 4.7% |

|  | <0.5 svgs/d | 0.5-<1 svgs/d | 1-<1.5 svgs/d | 1.5-<2 svgs/d | 2-<2.5 svgs/d | 2.5-<3 svgs/d | 3-<3.5 svgs/d | 3.5-<4 svgs/d | ≥4 svgs/d |
| --- | --- | --- | --- | --- | --- | --- | --- | --- | --- |
| 15-34 years | 2.8% | 15.2% | 23.2% | 20.6% | 15.2% | 9.9% | 5.9% | 3.3% | 4.0% |
| 35-54 years | 1.2% | 9.6% | 18.0% | 20.1% | 17.5% | 12.6% | 8.5% | 5.4% | 7.2% |
| 55-64 years | 1.4% | 10.6% | 18.8% | 19.7% | 17.3% | 12.7% | 8.0% | 5.1% | 6.6% |
| 65-74 years | 2.5% | 14.8% | 23.0% | 21.2% | 15.2% | 10.1% | 6.1% | 3.3% | 3.7% |
| ≥75 years | 2.9% | 15.6% | 23.3% | 21.2% | 15.2% | 9.5% | 5.6% | 3.2% | 3.5% |

Males

|  | <0.5 svgs/d | 0.5-<1 svgs/d | 1-<1.5 svgs/d | 1.5-<2 svgs/d | 2-<2.5 svgs/d | 2.5-<3 svgs/d | ≥3 svgs/d |
| --- | --- | --- | --- | --- | --- | --- | --- |
| ≤14 years | 17.0% | 32.4% | 23.2% | 13.3% | 7.1% | 3.6% | 3.4% |

|  | <0.5 svgs/d | 0.5-<1 svgs/d | 1-<1.5 svgs/d | 1.5-<2 svgs/d | 2-<2.5 svgs/d | 2.5-<3 svgs/d | 3-<3.5 svgs/d | 3.5-<4 svgs/d | 4-<4.5 svgs/d | 4.5-<5 svgs/d | ≥5 svgs/d |
| --- | --- | --- | --- | --- | --- | --- | --- | --- | --- | --- | --- |
| 15-34 years | 3.2% | 16.8% | 24.4% | 20.3% | 14.7% | 9.0% | 5.3% | 2.9% | 1.7% | 0.8% | 0.8% |
| 35-54 years | 1.4% | 10.7% | 19.2% | 20.7% | 16.8% | 12.2% | 7.8% | 4.8% | 2.9% | 1.6% | 2.0% |

|  | <0.5 svgs/d | 0.5-<1 svgs/d | 1-<1.5 svgs/d | 1.5-<2 svgs/d | 2-<2.5 svgs/d | 2.5-<3 svgs/d | 3-<3.5 svgs/d | 3.5-<4 svgs/d | ≥4 svgs/d |
| --- | --- | --- | --- | --- | --- | --- | --- | --- | --- |
| 55-64 years | 1.5% | 11.6% | 19.7% | 20.4% | 16.8% | 11.8% | 7.5% | 4.5% | 6.1% |
| 65-74 years | 2.9% | 16.7% | 23.1% | 21.2% | 15.2% | 9.2% | 5.5% | 2.9% | 3.3% |
| ≥75 years | 2.3 | 10.8 | 15.1 | 15.4 | 13.4 | 10.9 | 8.5 | 6.2 | 17.4 |

**Milk Servings (svgs)**

Females

|  | <0.5 svgs/d | 0.5-<1 svgs/d | 1-<1.5 svgs/d | 1.5-<2 svgs/d | ≥2 svgs/d |
| --- | --- | --- | --- | --- | --- |
| ≤14 years | 25.6% | 26.9% | 19.9% | 12.0% | 15.6% |
| 15-34 years | 49.0% | 25.0% | 12.8% | 6.4% | 6.9% |
| 35-54 years | 63.5% | 21.6% | 8.5% | 3.6% | 2.8% |
| 55-64 years | 67.3% | 20.3% | 7.7% | 2.7% | 2.1% |
| 65-74 years | 59.7% | 23.8% | 9.6% | 3.9% | 3.0% |
| ≥75 years | 57.9% | 24.6% | 10.0% | 4.2% | 3.4% |

Males

|  | <0.5 svgs/d | 0.5-<1 svgs/d | 1-<1.5 svgs/d | 1.5-<2 svgs/d | ≥2 svgs/d |
| --- | --- | --- | --- | --- | --- |
| ≤14 years | 21.4% | 23.7% | 19.8% | 14.0% | 21.1% |
| 15-34 years | 44.4% | 24.6% | 13.7% | 7.8% | 9.6% |
| 35-54 years | 58.5% | 21.9% | 10.2% | 4.8% | 4.5% |
| 55-64 years | 61.7% | 21.4% | 9.4% | 4.1% | 3.4% |
| 65-74 years | 53.1% | 24.6% | 11.5% | 5.7% | 5.1% |
| ≥75 years | 51.4% | 25.7% | 12.2% | 5.7% | 5.1% |

**Processed Meat (svgs)**

Females

|  | <0.25 svgs/d | 0.25-<0.5 svgs/d | ≥0.5 svgs/d |
| --- | --- | --- | --- |
| ≤14 years | 32.6% | 7.9% | 0.1% |

|  | <0.25 svgs/d | ≥0.5 svgs/d |
| --- | --- | --- |
| 15-34 years | 25.2% | 7.0% |
| 35-54 years | 19.6% | 4.2% |
| 55-64 years | 18.9% | 4.5% |
| 65-74 years | 18.8% | 4.1% |

|  | <0.25 svgs/d | 0.25-<0.5 svgs/d | ≥0.5 svgs/d |
| --- | --- | --- | --- |
| ≥75 years | 20.4% | 4.3% | 0.1% |

Males

|  | <0.25 svgs/d | 0.25-<0.5 svgs/d | ≥0.5 svgs/d |
| --- | --- | --- | --- |
| ≤14 years | 40.5% | 17.7% | 0.6% |

|  | <0.25 svgs/d | 0.25-<0.5 svgs/d | 0.5-<1 svgs/d | ≥1 svgs/d |
| --- | --- | --- | --- | --- |
| 15-34 years | 38.2% | 25.7% | 2.7% | 0.2% |

|  | <0.25 svgs/d | 0.25-<0.5 svgs/d | ≥0.5 svgs/d |
| --- | --- | --- | --- |
| 35-54 years | 36.2% | 19.7% | 1.7% |
| 55-64 years | 34.8% | 19.7% | 2.2% |
| 65-74 years | 35.3% | 19.4% | 2.1% |
| ≥75 years | 37.5% | 20.1% | 1.7% |

**Sugar Sweetened Beverages (svgs)**

Females

|  | 0.25<1 svgs/d | 1-<1.5 svgs/d | 1.5-<2 svgs/d | 2-<3 svgs/d | 3-<4 svgs/d | ≥4 svgs/d |
| --- | --- | --- | --- | --- | --- | --- |
| ≤14 years | 19.0% | 19.4% | 9.1% | 4.0% | 2.0% | 0.2% |

|  | 0.25<1 svgs/d | 1-<1.5 svgs/d | 1.5-<2 svgs/d | 2-<3 svgs/d | 3-<4 svgs/d | 4-<5 svgs/d | ≥5 svgs/d |
| --- | --- | --- | --- | --- | --- | --- | --- |
| 15-34 years | 36.2% | 10.9% | 6.1% | 5.8% | 2.0% | 0.6% | 0.2% |

|  | 0.25<1 svgs/d | 1-<1.5 svgs/d | 1.5-<2 svgs/d | 2-<3 svgs/d | 3-<4 svgs/d | 4-<5 svgs/d | ≥5 svgs/d |
| --- | --- | --- | --- | --- | --- | --- | --- |
| 35-54 years | 31.6% | 7.1% | 3.7% | 2.9% | 0.9% | 0.2% | 0.1% |

|  | 0.25<1 svgs/d | 1-<1.5 svgs/d | 1.5-<2 svgs/d | 2-<3 svgs/d | ≥3 svgs/d |
| --- | --- | --- | --- | --- | --- |
| 55-64 years | 27.6% | 5.4% | 2.6% | 1.9% | 0.6% |

|  | 0.25<1 svgs/d | 1-<1.5 svgs/d | 1.5-<2 svgs/d | 2-<3 svgs/d | ≥3 svgs/d |
| --- | --- | --- | --- | --- | --- |
| 65-74 years | 24.9% | 4.3% | 1.8% | 1.2% | 0.3% |

|  | 0.25<1 svgs/d | 1-<1.5 svgs/d | 1.5-<2 svgs/d | ≥2 svgs/d |
| --- | --- | --- | --- | --- |
| ≥75 years | 20.4% | 2.9% | 1.1% | 0.8% |

Males

|  | 0.25<1 svgs/d | 1-<1.5 svgs/d | 1.5-<2 svgs/d | 2-<3 svgs/d | 3-<4 svgs/d | 4-<5 svgs/d | ≥5 svgs/d |
| --- | --- | --- | --- | --- | --- | --- | --- |
| ≤14 years | 18.4% | 21.4% | 12.2% | 6.4% | 4.7% | 0.8% | 0.1% |

|  | 0.25<1 svgs/d | 1-<1.5 svgs /d | 1.5-<2 svgs/d | 2-<3 svgs/d | 3-<4 svgs/d | 4-<5 svgs /d | 5-<6 svgs /d | 6-<7 svgs /d | ≥7 svgs /d |
| --- | --- | --- | --- | --- | --- | --- | --- | --- | --- |
| 15-34 years | 35.6% | 13.4% | 9.2% | 10.7% | 4.6% | 1.8% | 0.6% | 0.2% | 0.1% |

|  | 0.25<1 svgs/d | 1-<1.5 svgs/d | 1.5-<2 svgs/d | 2-<3 svgs/d | 3-<4 svgs/d | 4-<5 svgs/d | 5-<6 svgs/d | ≥6 svgs/d |
| --- | --- | --- | --- | --- | --- | --- | --- | --- |
| 35-54 years | 35.6% | 10.4% | 6.5% | 6.4% | 2.4% | 0.7% | 0.3% | 0.1% |

|  | 0.25<1 svgs/d | 1-<1.5 svgs/d | 1.5-<2 svgs/d | 2-<3 svgs/d | 3-<4 svgs/d | 4-<5 svgs/d | ≥5 svgs/d |
| --- | --- | --- | --- | --- | --- | --- | --- |
| 55-64 years | 33.4% | 8.5% | 5.0% | 4.3% | 1.6% | 0.5% | 0.2% |

|  | 0.25<1 svgs/d | 1-<1.5 svgs/d | 1.5-<2 svgs/d | 2-<3 svgs/d | 3-<4 svgs/d | ≥4 svgs/d |
| --- | --- | --- | --- | --- | --- | --- |
| 65-74 years | 32.4% | 7.3% | 4.1% | 3.3% | 1.1% | 0.4% |

|  | 0.25<1 svgs/d | 1-<1.5 svgs/d | 1.5-<2 svgs/d | 2-<3 svgs/d | 3-<4 svgs/d |
| --- | --- | --- | --- | --- | --- |
| ≥75 years | 28.4% | 5.7% | 2.7% | 2.1% | 0.8% |

**Red Meat Servings (svgs)**

Females

|  | <0.5 svgs/d | 0.5-<1 svgs/d | ≥1 svgs/d |
| --- | --- | --- | --- |
| ≤14 years | 86.5% | 13.1% | 0.4% |
| 15-34 years | 60.9% | 39.0% | 0.1% |
| 35-54 years | 59.1% | 40.5% | 0.4% |
| 55-64 years | 55.6% | 43.6% | 0.8% |
| 65-74 years | 60.5% | 39.5% | 0.1% |
| ≥75 years | 77.5% | 22.5% | . |

Males

|  | <0.5 svgs/d | 0.5-<1 svgs/d | ≥1 svgs/d |
| --- | --- | --- | --- |
| ≤14 years | 77.9% | 21.0% | 1.1% |
| 15-34 years | 24.8% | 54.2% | 21.0% |
| 35-54 years | 23.7% | 52.5% | 23.9% |

|  | <0.5 svgs/d | 0.5-<1 svgs/d | 1-<1.5 svgs/d | ≥1.5 svgs/d |
| --- | --- | --- | --- | --- |
| 55-64 years | 21.7% | 51.2% | 27.1% | 0.1% |

|  | <0.5 svgs/d | 0.5-<1 svgs/d | ≥1 svgs/d |
| --- | --- | --- | --- |
| 65-74 years | 23.9% | 54.4% | 21.8% |
| ≥75 years | 38.7% | 54.5% | 6.8% |
